# Implications of Genetic Factors Underlying Mouse Hydronephrosis: Cautionary Considerations on Phenotypic Interpretation in Genetically Engineered Mice

**DOI:** 10.3390/ijms25137203

**Published:** 2024-06-29

**Authors:** Shino Nemoto, Kazuyo Uchida, Hiroshi Ohno

**Affiliations:** 1 Laboratory for Intestinal Ecosystem, RIKEN Center for Integrative Medical Sciences, Yokohama 230-0045, Japan; 2 Laboratory for Immune Regulation, Graduate School of Medical and Pharmaceutical Sciences, Chiba University, Chiba 260-0856, Japan; 3 Immunobiology Laboratory, Graduate School of Medical Life Science, Yokohama City University, Yokohama 230-0045, Japan

**Keywords:** hydronephrosis, mouse model, kidney, liver, X chromosome, ornithine transcarbamylase

## Abstract

Hydronephrosis, the dilation of kidneys due to abnormal urine retention, occurs spontaneously in certain inbred mouse strains. In humans, its occurrence is often attributed to acquired urinary tract obstructions in adults, whereas in children, it can be congenital. However, the genetic factors underlying hydronephrosis pathogenesis remain unclear. We investigated the cause of hydronephrosis by analyzing tetraspanin 7 (*Tspan7*) gene-modified mice, which had shown a high incidence of hydronephrosis-like symptoms. We found that these mice were characterized by low liver weights relative to kidney weights and elevated blood ammonia levels, suggesting liver involvement in hydronephrosis. Gene expression analysis of the liver suggested that dysfunction of ornithine transcarbamylase (OTC), encoded by the X chromosome gene *Otc* and involved in the urea cycle, may contribute as a congenital factor in hydronephrosis. This OTC dysfunction may be caused by genomic mutations in X chromosome genes contiguous to *Otc*, such as *Tspan7*, or via the genomic manipulations used to generate transgenic mice, including the introduction of Cre recombinase DNA cassettes and cleavage of loxP by Cre recombinase. Therefore, caution should be exercised in interpreting the hydronephrosis phenotype observed in transgenic mice as solely a physiological function of the target gene.

## 1. Introduction

Genetically homogenous inbred laboratory mice are essential resources for functional gene analyses. However, various spontaneous pathologies in laboratory mice [1,2], such as nephropathy, glomerulonephritis, acidophilic macrophage pneumonia, tumors, ulcerative dermatitis, myocarditis, fatty liver, testicular atrophy, ovarian atrophy, pituitary cysts, adrenal pigmentation, and hematopoietic tumors such as lymphomas, can cause confusion in interpreting mouse phenotypes resulting from animal experimentation. Kidney-related diseases are the second leading cause of death in laboratory mice [3], making it especially important to exercise caution when interpreting the phenotypes of experimental mice.

Hydronephrosis, a condition in which the kidneys dilate due to abnormal urine retention, occurs spontaneously in some inbred strains of laboratory mice [4,5,6,7,8,9]. Given the interstrain variation in its incidence, genetic factors are speculated to be involved in the development of hydronephrosis. In severe cases of hydronephrosis in humans, the renal parenchyma may be damaged, leading to renal failure that requires dialysis or kidney transplantation [10]. In adults, 70–80% of hydronephrosis cases are attributed to acquired urinary tract obstruction from various causes [11,12]. However, its occurrence in approximately 1% of births [13,14] indicates the involvement of congenital factors [15,16]. Furthermore, males are twice as likely to develop hydronephrosis as females [11], suggesting a hitherto unknown genetic predisposition to the disease. In mice, abnormalities in renal function, including electrolyte transporters, water channels and pumps, the renin-angiotensin system, and dysfunctions in renal cell differentiation and proliferation, are involved in the development and progression of hydronephrosis [17]. Various genes, including *Fcgr* [18], *Cph* [17], *Wntb* [19], *Wnt7b* [20], *p23* [21], *Tcf21* [22], *Osr1* [23], *miR-143/145* [24], *MRL* [25], and *Id2* [26], are involved in the pathology of this disease. Mouse models of hydronephrosis, in which hydronephrosis is induced chemically, e.g., by administering 2,3,7,8-tetrachlorodibenzo-p-dioxin [27], which activates aryl hydrocarbon receptor signaling [28], or physically, e.g., by ureteral ligation [29,30], have been created. Although numerous theories have been proposed to explain the pathogenesis of hydronephrosis, the causative genes and genetic predispositions have not been elucidated.

Male mice exhibit a higher susceptibility to hydronephrosis [4,5,7], with the right kidney often being more severely affected than the left kidney [4,5,6,7] and with the development of dimples inside the kidney due to urinary retention [5,7,8]. Recently, while conducting a study on obesity, we encountered a hydronephrosis-like phenomenon in genetically engineered mice expressing Cre recombinase under the adiponectin (*Adipoq*) promoter, targeting tetraspanin 7 (*Tspan7*) in an adipose-tissue-specific manner on a C57BL/6 background [31]. *Tspan7* is known to be involved in cell signaling and interaction processes that influence various physiological functions, including cell proliferation, migration, and tissue organization [32,33]. However, it remained unclear whether the observed hydronephrosis-like phenomenon was a manifestation of *Tspan7* function or a spontaneous lesion in inbred mice. Because the characteristics of the dilated kidneys frequently observed in *Tspan7* transgenic mice fit the pathomorphological definition of hydronephrosis lesions [10], in the present study, we attempted to identify the characteristics and causes of abnormal kidneys and the molecular mechanisms underlying hydronephrosis in these mice and contribute to the understanding of this condition.

## 2. Results

### 2.1. Discovery of Tspan7 Transgenic Mice with Kidney Abnormalities Resembling Hydronephrosis

**Figure 1:**
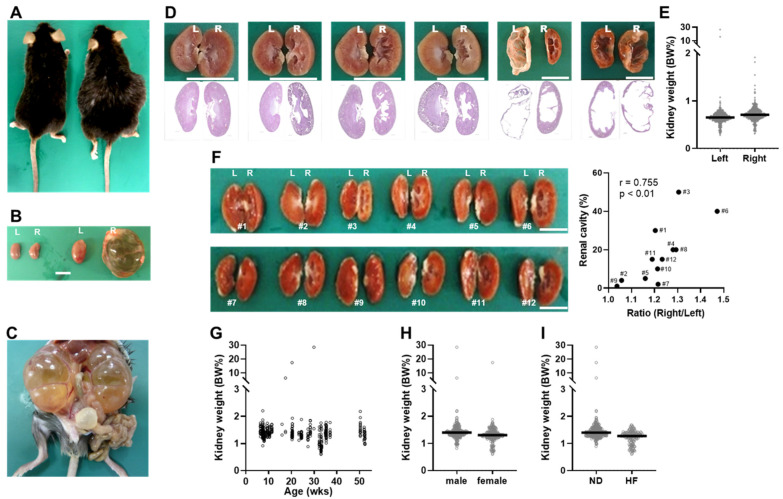
Hydronephrosis-like pathology observed in *Tspan7* transgenic mice. (**A**) Appearance of a mouse with normal kidneys (on the left) and a mouse with dilated kidneys (on the right) and (**B**) their left (L) and right (R) kidneys. The animals were a 30-week-old male adipocyte-specific knockout (AKO) mouse (right) and its littermate (left, AKO flox/flox). (**C**) Severely dilated kidneys in a 15-week-old male AKO mouse. (**D**) Bisected left and right kidneys (upper panel) and corresponding hematoxylin and eosin (H&E)-stained sections (lower panel). From left to right, kidneys from an 8-week-old male AKO flox/flox, 35-week-old male AKO, 35-week-old male AKO flox/flox, 15-week-old female AOE flox/flox, 20-week-old female AOE, and 17-week-old male AOE mouse are shown. (**E**) Left and right kidney weights per body weight (BW%) of male (*n* = 256: AKO = 81, AKO flox/flox = 89, AOE = 39, AOE flox/flox =47) and female (*n* = 224: AKO = 67, AKO flox/flox = 84, AOE = 36, AOE flox/flox =37) mice from 10 to 52 weeks of age. (**F**) Images of left and right kidneys (right panel) and correlation between the percentage of renal cavities and the ratio of right kidney weight to left kidney weight. The numbers in the image correspond to those in the correlation chart. The images and data were obtained from 15-week-old male AKO (#1, #2, #3, #4, #6, #7, #8, #10, and #11) and AKO flox/flox (#5, #9, and #12) mice. (**G**–**I**) Distribution of kidney weight (BW%) by age (**G**), sex (*n* = 480: male = 256, female = 224) (**H**), and diet (*n* = 480: ND = 340, HF = 140) (**I**). The plots show individual data. Black lines show the median. Thes statistical significance among groups was assessed using the Mann–Whitney *U* test. White line, 10 mm. AOE, adipocyte-specific *Tspan7*-overexpressing mice; AOE flox/flox, AOE littermates; ND, normal diet; HF, high-fat diet.

In our previous study on obesity using *Tspan7* transgenic mice [31], dilated kidneys were frequently observed in the mice. In mice with severely dilatated kidneys, it was possible to infer that the kidneys were dilated from the bulges in their flank even without dissection (Figure 1A,B). Some mice had only one kidney dilated, whereas in others, both kidneys were dilated (Figure 1C). Among the 480 mice used in this study, one mouse had no left kidney, and its right kidney was dilated. The appearance of kidney abnormalities varied; in some cases, the kidneys were enlarged so much that the renal blood vessels became clearly visible (Figure 1B,C), whereas in others, internal cavities of various sizes were visible upon bisecting the kidney (Figure 1D). When the enlarged kidney was split in half, the fluid that came out was yellowish and ammonia was detected. Hematoxylin and eosin (H&E)-stained kidney section images revealed structural collapse of the kidney parenchyma, including atrophy and dilation of the renal pelvis (Figure 1D), which is responsible for draining urine into the ureter [10]. Dilation often appeared in the right kidney, although a giant left kidney was also occasionally present (Figure 1D). The right kidney weighed significantly more than the left (*p* < 0.0001; Figure 1E). Upon separating the kidneys into halves, the heavier the right kidney was compared to the left kidney, the more internal cavities were observed (Figure 1F). Kidney dilation was not associated with age (Figure 1G). However, there were differences according to sex and diet; males had heavier kidneys (BW%) than females (*p* < 0.0001, Figure 1H), and normal diet (ND)-fed mice had heavier kidneys (BW%) than high-fat diet (HF)-fed mice (*p* < 0.01, Figure 1I).

### 2.2. Mice with Dilated Kidneys Exhibit Hyperammonemia and Atrophic Liver

To characterize the mice with hydronephrosis-like pathology, we first divided them into groups with enlarged and normal kidneys (hereafter referred to as “large” and “normal”) based on kidney size, which was defined as the percentage of kidney weight per body weight (kidney BW%), and compared the groups. High absolute kidney weights did not necessarily reflect large kidneys, as they correlated with body weight (Spearman’s r = 0.87, *p* < 0.0001, Figure S1A). The kidneys in males were heavier than those in females (Figure S1A), and their weight increased with age (Figure S1B). Therefore, kidney BW%, which did not correlate with age (Figure 1G), was used in the analysis. Because the 95% confidence interval (CI) of the mean kidney BW% for all 480 samples ranged from 1.3263% to 1.5907%, we considered mice with a kidney BW% ≥1.5907% to have dilated kidneys. We determined the characteristics of mice with hydronephrosis-like pathology by comparing these mice with those having a kidney BW% <1.5907% (normal-kidney mice).

**Figure 2:**
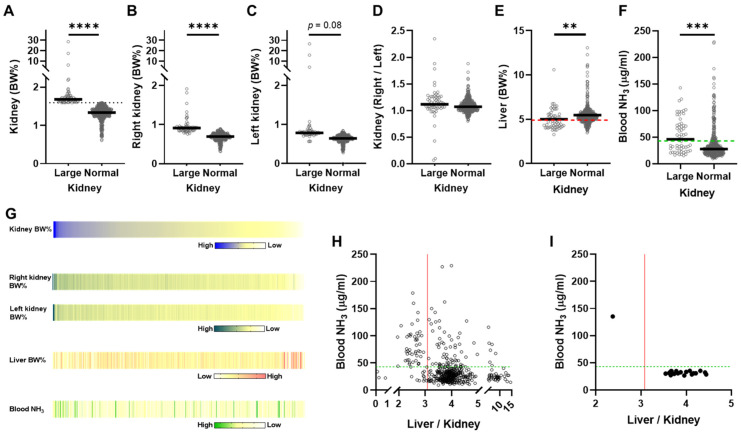
Characteristic phenotypes and number of mice with hydronephrosis-like pathology. (**A**) Male (*n* = 256: AKO = 81, AKO flox/flox = 89, AOE = 39, AOE flox/flox = 47) and female (*n* = 224: AKO = 67, AKO flox/flox = 84, AOE = 36, AOE flox/flox = 37) mice aged 10–52 weeks (total *n* = 480) were divided into large- and normal-kidney groups according to the upper limit of the mean CI for kidney BW% (1.5907, dotted line). (**B**) Right and (**C**) left kidney weights (BW%), (**D**) ratio of right to left kidney weight, and (**E**) liver weight (BW%) in the large- and normal-kidney groups. The red dotted line (4.902) indicates the 25th quartile. (**F**) Blood ammonia concentrations in the large- and normal-kidney groups. The green dotted line (41.7) indicates the upper limit of the 95% CI for the mean ammonia concentration. (**G**) Heatmap of the weights of both kidneys, right kidney, left kidney, and liver and the blood ammonia concentration in the 480 mice ordered from high to low kidney BW%. (**H**) Relationship between the weight liver/kidney ratio and the blood ammonia concentration. The red line (3.081) indicates the 25th quartile of liver BW% (4.902)/upper limit of 95% CI for the mean kidney BW% (1.5907). (**I**) Relationship between the liver-to-kidney weight ratio and the blood ammonia concentration in 15-week-old intact male mice (*n* = 20). Large, mice with kidney BW% ≥ 1.5907; normal, mice with kidney BW% < 1.5907. Black lines indicate medians. The statistical significance of differences among groups was assessed using the Mann–Whitney *U* test. **, *p* < 0.01; ***, *p* < 0.001; ****, *p* < 0.0001; CI, confidence interval; AKO, adipocyte-specific *Tspan7*-knockout mice; AKO flox/flox, AKO littermates; AOE, adipocyte-specific *Tspan7*-overexpressing mice; AOE flox/flox, AOE littermates.

Of the 480 mice, 59 (12.3%) were categorized as having large kidneys (Table 1 and Figure 2A). Both kidneys were larger in the large-kidney group than in the normal-kidney group. However, the difference was more pronounced in the right kidney than in the left kidney (*p* < 0.0001 and *p* = 0.08, respectively; Figure 2B,C). The phenotype of an enlarged right kidney was observed in both the large- and normal-kidney groups, with no significant difference between the two groups (84.7% and 80.7%, respectively; Figure 2D). The data suggested a relation between right kidney enlargement and kidney dilation phenomenon. As numerous reports indicate that the right kidney is larger than the left kidney [4,5,6,7,23], we hypothesized that the location of the liver above the right kidney might be responsible for the physiological changes, and therefore, we first examined the size of the liver in both groups.

The liver tissue weight (liver BW%) in the large-kidney mouse group was significantly (*p* < 0.01, Figure 2E) lower than that in the normal-kidney mouse group. Mice with a 25th percentile of liver BW% of ≤ 4.902% (small-liver mice) accounted for 52.5% (31/59 mice, Table 1) of large-kidney mice, whereas among normal-kidney mice, they comprised only 21.1% (89/421 mice, Table 1). The odds ratio of small-liver mice appearing among large-kidney mice was 4.13 (95% CI: 2.31 to 7.13, *p* < 0.0001, Fisher’s exact test). These results suggest that the liver is involved in or affected by the development and progression of kidney dilation in these mice.

The liver converts nitrogen compounds such as ammonia into urea, which is excreted as urine by the kidneys, indicating a close relationship between the kidneys and liver. Therefore, to determine whether the kidney dilation observed was a result of abnormal liver function, we measured blood ammonia levels in mice in the large- and normal-kidney groups. The blood ammonia concentrations in the large-kidney group were significantly higher than those in the normal-kidney group (*p* < 0.001, Figure 2F). Furthermore, 52.6% (29 + 2 = 31/59 mice, Table 1) of mice in the large-kidney group had ammonia concentrations ≥41.7 μg/mL (the upper limit of the 95% CI for the mean ammonia concentrations), whereas only 22.8% (42 + 54 = 96/421 mice, Table 1) of mice in the normal-kidney group had concentrations above this level. Notably, 29 (93.5%) of the 31 mice with large kidneys and small livers had high levels of ammonia in their blood, whereas only two of the 28 (7.1%, Table 1) large-kidney mice with normal livers had high blood ammonia levels. Even in normal-kidney mice, 47.2% (42/89 mice, Table 1) of mice with small livers had high blood ammonia levels.

The values of kidney weight, liver weight, and blood ammonia concentration for the 480 *Tspan7* transgenic mice are shown in Figure 2G. Mice with larger kidneys often had smaller livers and higher blood ammonia levels. However, mice with large kidneys did not necessarily have high blood ammonia levels, nor did mice with high blood ammonia levels necessarily have large kidneys. Mice with small livers persistently exhibited hyperammonemia, but those with hyperammonemia did not necessarily have smaller livers. We found that mice with lower liver weights relative to kidney weights had higher blood ammonia levels (Figure 2H).

The previously described three-way relationship (Figure 2H) was also observed in intact C57BL/6J mice (20 male mice fed a normal diet from 6 to 15 weeks of age), the C57BL/6 substrain being used as background for generating the *Tspan7* transgenic mice (Figure 2I). As inbred C57BL mice spontaneously develop kidney dilation [1], the kidney dilation observed in the transgenic mice may merely be a spontaneously occurring pathology. Therefore, we examined the incidence of large kidneys (kidney BW% ≥ 1.5907), small liver (liver BW% ≤ 4.902), and a high blood ammonia level (≥41.7) in background mice (Figure S2A–F). Only one mouse (5%) had large kidneys (Figure S2B), and this was the only mouse with a small liver (Figure S2E) and high blood ammonia level (Figure S2F). No significant difference was observed between the right and left kidneys (Figure S2C,D). To determine whether the development of a hydronephrosis-like pathology in *Tspan7* transgenic mice is due to spontaneous development in inbred mice or depends on an additional developmental component due to genetic engineering, we calculated the odds ratio (OR) by comparing the incidence of mice with both large kidneys and a small liver between *Tspan7* transgenic and intact mouse groups. The odds of observing this particular phenotype in *Tspan7* transgenic mice were seven times higher than those for intact mice (OR = 7.397, 95% CI: 1.240–78.69, *p* = 0.029, Table S1). This suggested that the development of hydronephrosis-like pathology in *Tspan7* transgenic mice occurred as a result of genetic manipulation, as well as spontaneously.

### 2.3. The Incidence of Hydronephrosis-like Pathology Is High in Genetically Engineered Mice

**Figure 3:**
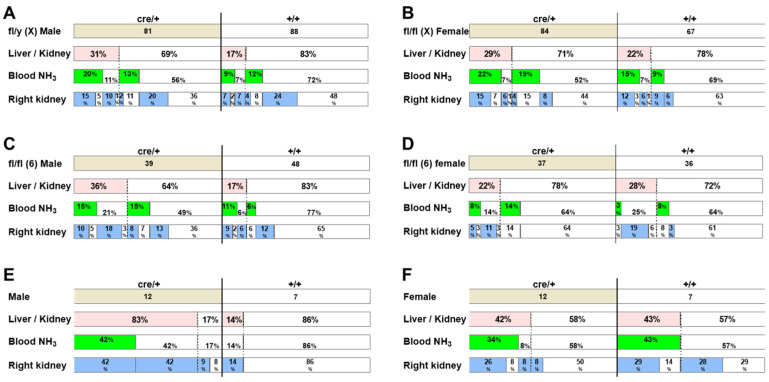
High incidence of hydronephrosis-like pathology in Cre-incorporated mice. The numbers inside the upper columns represent the numbers of mice analyzed for (**A**) fl/y(X) male, (**B**) fl/fl(X) female, (**C**) fl/fl(6) male, (**D**) fl/fl(6) female, (**E**) *Adipoq*-Cre male, and (**F**) *Adipoq*-Cre female mice. Pink column (Liver/Kidney): incidence of mice with a liver-to-kidney weight ratio < 3.081; green column (Blood NH_3_): incidence of mice with a blood ammonia concentration > 42.9 µg/mL; blue column (Right kidney): incidence of mice with a right kidney-to-body weight ratio >0.85. fl/y(X) and fl/fl(X), *Tspan7*-knockout mice; fl/fl(6), *Tspan7*-overexpressing mice; cre/+, Cre-positive mice; +/+, Cre-negative mice.

To investigate the reason for the higher incidence of hydronephrosis-like pathology in *Tspan7* transgenic mice compared to intact mice, we analyzed the incidence of abnormal phenotypes induced by genomic manipulation during the process of generating transgenic mice (Figure 3A–D and Table S2). We examined 480 transgenic mice from two lines [31] with different levels of *Tspan7* expression. One line was *Tspan7*-knockout mice, and the other line was *Tspan7*-pverexpressing mice. We assumed that homologous recombinant mice with *Tspan7* located on the X chromosome—fl/y(X) and fl/fl(X)—would have a DNA cassette with loxP integrated into chromosome X (*Tspan7*-knockout mice), and transgenic mice with fl/fl(6) in the *Rosa26* locus on chromosome 6 would have a DNA cassette with loxP integrated into chromosome 6 (*Tspan7*-pverexpressing mice). Because both mouse lines have loxP sequences in the incorporated DNA, when crossed with mice having a cassette containing a Cre recombinase sequence downstream of the *Adipoq* promoter, *Tspan7* expression is altered in a Cre-dependent manner. Accordingly, the 480 mice analyzed comprised four different genomic patterns: [fl/y(X) or fl/fl(X), cre/+], [fl/y(X) or fl/fl(X), +/+], [fl/fl(6), cre/+], and [fl/fl(6), +/+]. Figure 3 shows the incidence of hydronephrosis-like pathologies in these different genomic groups of mice for males and females separately. The data indicate that Cre integration may cause hydronephrosis-like pathology. For example, among fl/y(X) male mice (Figure 3A), the incidence of mice with both large kidneys and a small liver was 17% (15 of 88 mice) in +/+ mice, whereas it was 31% (25 of 81 mice) in mice with integrated Cre (cre/+). The incidence of this phenotype in cre/+ mice was twice as high as that in +/+ mice (OR = 2.173, 95% CI: 1.044–4.593, *p* = 0.046, Table S2). Furthermore, the incidence of mice with a high blood ammonia level was 35% (28 of 81 mice) in cre/+ mice and >21% (18 of 88 mice) in +/+ mice (OR = 1.966, 95% CI: 1.006–3.790, *p* = 0.059, Table S2). The higher incidence of hydronephrosis-like symptoms such as large kidneys, a small liver, and a high blood ammonia level in cre/+ mice than that in +/+ mice was observed not only in fl/y(X) male mice but also in fl/fl(X) female mice (Figure 3B and Table S2) and fl/fl(6) male mice (Figure 3C and Table S2). The fact that the same phenotype appeared in both *Tspan7*-knockout fl/y(X)/fl/fl(X) and *Tspan7*-overexpressing fl/fl(6) mice suggests that a hydronephrosis-like pathology was not due to Cre-dependent *Tspan7* transcript levels but may have been related to the action of Cre transcripts or the integration of Cre-containing DNA cassettes.

**Figure 4:**
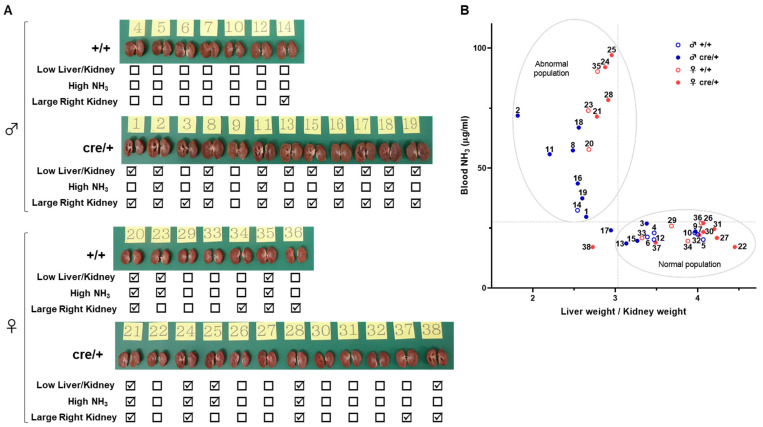
*Adipoq*-Cre mice also exhibit hydronephrosis-like pathology. (**A**) Bisected left and right kidneys from 15-week-old *Adipoq*-Cre male (*n* = 19) and female (*n* = 19) mice. ☑ marked: Low Liver/Kidney, a liver-to-kidney weight ratio < 3.081; High NH_3_, a blood ammonia concentration > 41.7 µg/mL; Large Right Kidney, a right kidney-to-body weight ratio > 0.73. (**B**) Plot of liver-to-kidney weight ratio versus blood ammonia concentration. The numbers assigned to each dot corresponds to the number in (**A**). The population with low liver-to-kidney weight ratios (<3.081) and high blood ammonia levels was designated “Abnormal”. cre/+, Cre-positive mice; +/+, Cre-negative mice; blue open circles, male +/+ mice; blue closed circles, male cre/+ mice; red open circles, female +/+ mice; red closed circles, female cre/+ mice.

If integration of the Cre expression cassette would be responsible for the hydronephrosis-like pathology, the pathology should be observed in the Cre-expressing mouse line used to generate the conditional mutant mice, even without breeding with mice carrying the loxP-*Tspan7* DNA. We examined the incidence of hydronephrosis-like pathology in *Adipoq*-Cre mice used to generate *Tspan7* transgenic mice (Figure 3E,F and Table S2). Eighty-three percent (10 of 12) of male cre/+ mice had large kidneys and a small liver, and half of these (5 of 10 mice) showed a high blood ammonia level (Figure 3E). In contrast, in +/+ mice, 14% (1 of 7 mice) had both a large kidney and a small liver, but none of these showed a high blood ammonia level (Table S2). Furthermore, among male mice, more cre/+ mice than +/+ mice had a large right kidney (92%: 11/12 mice vs. 14%: 1/7 mice, *p* = 0.002, Table S2). In contrast, among female mice, no difference in the incidence of hydronephrosis-like phenotypic features was noted between +/+ and cre/+ mice (Figure 3F and Table S2). However, cavities inside the kidneys, presumably caused by gouging due to urine retention, appeared in female as well as male cre/+ mice (Figure 4A). In addition, the relationship between lower liver weights relative to kidney weights and higher blood ammonia levels observed in *Tspan7* transgenic mice (Figure 2H) was also observed in both male and female *Adipoq*-Cre mice (Figure 4B). Taken together, these results suggested that Cre expression cassette integration may be responsible for a hydronephrosis-like pathology, in addition to spontaneous development in inbred mice.

### 2.4. X Chromosome Mutations May Cause Hydronephrosis-like Pathology

We questioned how Cre-expression cassette integration would cause renal abnormalities and speculated that the X chromosome might be involved in hydronephrosis-like phenotypes based on our observations and the location of *Tspan7* on the chromosome. That is, males were more susceptible to hydronephrosis-like conditions than females (Figure 3); the increased incidence of hydronephrosis-like pathology caused by Cre integration was not observed in fl/fl(6) female mice (Figure 3D and Table S2) but was observed in fl/y(X) mice (Figure 3B and Table S2), and ornithine transcarbamylase (*Otc*), one of the genes responsible for the urea cycle that converts ammonia to urea, is adjacent to *Tspan7*. In humans, mutations or deletions in *OTC* cause a genetic disorder known as OTC deficiency (OTCD; OMIM 300461). Patients with this disease develop clinical symptoms of the central nervous system because of urea cycle impairment, which results in the accumulation of ammonia in the body [34]. Genomic analysis of patients with OTCD have revealed the deletion of TSPAN7 [35,36,37]. Therefore, we speculated that the genome around *Tspan7* was no longer intact in *Tspan7* transgenic mice, and *Otc* was affected, which might have affected *Otc* expression and, in turn, the physiological function of its protein product. The high blood ammonia levels in *Tspan7* transgenic mice (Figure 2 and Figure 3, Table 1 and Table S2) may be the consequence of a defect in the urea cycle due to dysfunctional OTC. Therefore, to determine whether the hydronephrosis-like pathology frequently observed in this study was related to *Otc*, we examined the gene expression profiles of the liver, which is the primary OTC-expressing organ that carries out the urea cycle.

### 2.5. Otc and Urea Cycle Gene Expression Profiles in Mice with Hydronephrosis-like Pathology

**Figure 5:**
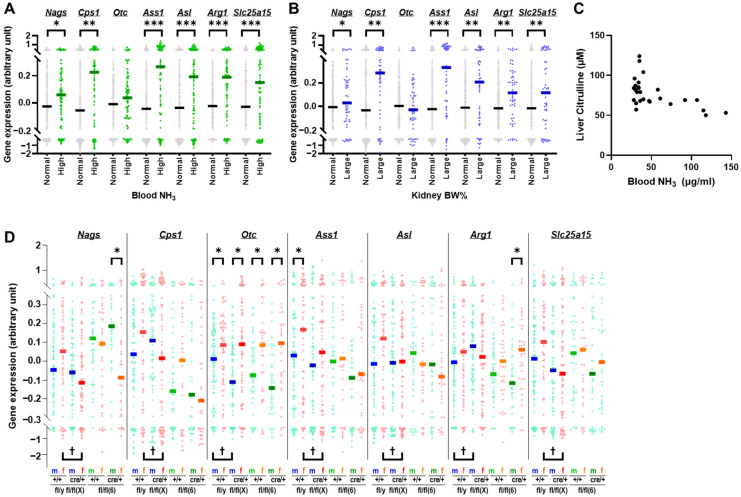
The liver expression profile of *Otc* differs from that of other urea cycle genes. Comparison of urea cycle gene expression levels between (**A**) mice with high (*n* = 104) and normal (*n* = 376) blood ammonia levels and (**B**) mice with large (*n* = 54) and normal (*n* = 426) kidneys. The statistical significance of the difference among groups was assessed using the Mann–Whitney *U* test. * *p* < 0.05, ** *p* < 0.01, and *** *p* < 0.001. (**C**) Liver citrulline and blood ammonia levels. (**D**) Expression of urea cycle genes in male and female mice, fl/y(X), fl/fl(X), and fl/fl(6) mice, with and without Cre incorporation. The lines indicate the median, and the plots show individual data: blue, male fl/y(X), *n* = 170; red, female fl/fl(X), *n* = 151; green, male fl/fl(6), *n* = 86; orange, female fl/fl(6), *n* = 73. m, male; f, female; fl/y(X) and fl/fl(X), *Tspan7*-knockout mice; fl/fl(6), *Tspan7*-overexpressing mice; cre/+, Cre-positive mice; +/+, Cre-negative mice. The statistical significance of the difference among groups was assessed using the Mann–Whitney *U* test. * *p* < 0.05 between male and female mice within the same mouse line and genotype; †, *p* < 0.05 between +/+ and cre/+ groups within the same mouse line and sex.

Figure 5 shows the expression levels of seven urea cycle genes, N-acetylglutamate synthase (*Nags*), carbamylphosphate synthetase (*Cps1*), *Otc*, argininosuccinate synthetase (*Ass1*), argininosuccinate lyase (*Asl*), arginase (*Arg1*), and solute carrier family 25 member 15 (*Slc25a15*) in the liver.

In mice with high blood ammonia levels (>41.7 μg/mL), the expression levels of all urea cycle genes, except *Otc*, were significantly higher than those in normal mice (≤41.7 μg/mL) (*p* < 0.05, Figure 5A). Similarly, in mice with large kidneys (>1.5907 BW%), the expression levels of all urea cycle genes except *Otc* were significantly higher than those in normal mice (≤1.5907 BW%) (*p* < 0.05, Figure 5B). Thus, *Otc* expression was not induced in mice with hydronephrosis-like pathological features, such as high blood ammonia levels and large kidneys, suggesting that it may be defective in these mice. The functional defect in OTC was corroborated by the lower concentration of citrulline, a product of the reaction catalyzed by OTC, in the livers of mice with higher blood ammonia levels (Figure 5C).

Figure 5D shows the expression levels of urea cycle genes with or without Cre integration (cre/+ or +/+) in male and female mice for each chromosome in which a DNA cassette containing loxP was incorporated (fl/y(X) and fl/fl(X) or fl/fl(6)). The expression of *Otc*, the only urea cycle gene on the X chromosome, was significantly lower in males than in females after any DNA manipulation (*p* < 0.05); such lower expression was not observed for urea cycle genes located on other chromosomes (*Nags*: Chr 11, *Cps1* Chr 1, *Ass1*: Chr 2, *Asl:* Chr 5, and *Slc25a15*: Chr 8). This indicates that mutations in genes on the male X chromosome, of which there is only one, affect transcription, whereas mutations on the female X chromosome, of which there are two, compensate for each other. Furthermore, significant alterations in urea cycle gene expression levels due to Cre integration were observed only in fl/(X) mice (*Nags*, *Cps1*, *Ass1*, *Asl*, and *Slc25a15* in female mice fl/fl(X) and *Otc* and *Arg1* in male mice fl/y(X), *p* < 0.05, Figure 5D), not in fl/fl(6) mice. These results suggested that mutations in genes on the X chromosome are involved in the hydronephrosis-like pathology observed in *Tspan7* transgenic mice.

### 2.6. Expression Levels of Genes Adjacent to Otc in Mice with Hydronephrosis-like Pathology

**Figure 6:**
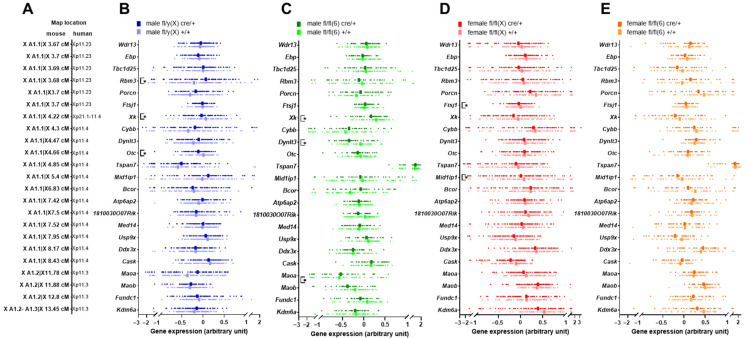
Expression profiles of genes adjacent to *Otc* on the X chromosome. (**A**) Location of mouse and human genes. Indicated gene expression levels in the liver of (**B**) fl/y(X) males (*n* = 170: cre/+ = 81, +/+ = 89), (**C**) fl/fl(6) males (*n* = 86: cre/+ = 39, +/+ = 47), (**D**) fl/fl(X) females (*n* = 151: cre/+ = 84, +/+ = 67), and (**E**) fl/fl(6) females (*n* = 73: cre/+ = 37, +/+ = 36). The lines indicate medians and the plots show individual data. The statistical significance of differences among groups was assessed using the Mann–Whitney *U* test. *, *p* < 0.05 between +/+ and cre/+ groups within the same mouse line and sex.

Genomic deletions and mutations that extend across multiple genes result in contiguous gene syndrome symptoms. We surmised that if manipulation of *Tspan7* triggered X-linked contiguous gene syndromes and *Tspan7* transgenic mice developed OTCD-like symptoms such as hyperammonemia, the expression levels of genes other than *Otc* may also be affected. Therefore, we examined the expression levels of genes around X A1.1 (corresponding to human Xp11.4, Figure 6A), where *Otc* and *Tspan7* are located, with respect to Cre integration (cre/+ or +/+), the chromosome in which the DNA cassette containing the loxP sequence integrates (fl/(X) or fl/fl(6)), and sex (Figure 6B–E).

In fl/y(X) male mice, in which *Otc* expression was significantly reduced upon Cre integration, the expression of the X-linked Kx blood group antigen (*Xk*) was significantly reduced (*p* < 0.05, Figure 5B). A significant reduction in *Xk* expression upon Cre integration was also observed in the fl/fl(6) male mice (*p* < 0.05, Figure 6C). In addition, in fl/y(X) male mice, the expression of the RNA-binding motif (RNP1, RRM) protein 3 gene (*Rbm3*) was significantly increased (*p* < 0.05), and in fl/fl(6) male mice, the expression of the genes encoding dynein light chain Tctex-type 3 (*Dynlt3*), monoamine oxidase B (*Maob*), and FUN14 domain containing 1 (*Fundc1*) was significantly decreased (*p* < 0.05, Figure 6B,C). In fl/fl(X) female mice, the gene expression levels of FtsJ RNA 2′-O-methyltransferase 1 (*Ftsj1*) and MID1-interacting protein 1 (*Mid1ip1*) were significantly reduced (*p* < 0.05, Figure 6D), whereas none of the genes were affected in fl/fl(6) female mice (Figure 6E).

*Xk* (Figure 6B,C), *Dynlt3* (Figure 6C), and *Mid1ip1* (Figure 6D) are deleted along with OTC in patients with OTCD [38]. Their transcription was downregulated in mice with hydronephrosis-like pathology, suggesting that X-linked contiguous syndrome may occur in *Tspan7* transgenic mice. However, we cannot decisively conclude that *Tspan7* manipulation causes contiguous syndrome because we did not identify any genomic deletion regions or breakpoints in *Tspan7* transgenic mice; nor did we confirm that the expression levels of genes adjacent to *Otc* in *Tspan7* transgenic mice were reduced compared with those in intact mice without genome manipulation.

## 3. Discussion

In our investigation of *Tspan7* transgenic mice aimed at understanding the physiological functions of *Tspan7* [31], we consistently observed abnormalities in the kidneys, characterized by an unusual bulging appearance and a spongy texture (Figure 1). Nevertheless, the abnormalities did not appear to compromise viability, as mice did not die even when their kidneys were severely enlarged. Upon bisecting the abnormal kidneys, we noticed a yellow liquid with extremely high ammonia content oozing out of the interior and the existence of internal cavities (Figure 1D–F). Histological analysis revealed a disproportionate increase in kidney weight compared to parenchymal tissue (Figure 1D), suggesting that the kidney bulging and increased weight were primarily due to urine accumulation. Furthermore, elevated blood ammonia levels (Figure 2F,G) and decreased expression of genes encoding major urinary proteins (*Mup1*, *2*, *3*, *4*, *5*, *6*, *7*, *8*, *9*, *10*, *11*, *12*, *13*, *14*, *16*, *17*, *18*, *19*, *21*, FC > 1, *p* < 0.05, Figure S3) indicated a disruption of the urinary excretion mechanisms, reminiscent of the pathogenesis of hydronephrosis.

Hydronephrosis in humans is often caused by obstruction of the urinary tract caused by ureteral stones, tumors, ureteral polyps, and benign prostatic hyperplasia or strictures of the urinary flow pathways, such as the connection between the kidney and ureter, the ureter and bladder, or urethral valves [10,11]. Hence, the abnormal kidneys observed in *Tspan7* transgenic mice should have required an anatomical examination of the renal pelvis, glomeruli, tubules, wall cysts, cysts, renal portals, and ureters to determine whether any of these locations obstructed the urinary tract. However, given that hydronephrosis in children is often congenital [13,14,15,16] and that, in some mouse strains, it develops spontaneously [4,5,6,7,8,9], we first sought a genetic predisposition for this condition.

Our finding that mice with a liver-to-kidney weight ratio of ≤3.08 had high blood ammonia levels (Figure 2H,I and Figure 4B), which is indicative of hydronephrosis, suggested that the liver was involved in hydronephrosis in these mice. To determine whether the liver in mice with hydronephrosis-like pathology was inflamed or damaged, we examined blood markers of hepatocellular damage, including alanine aminotransferase (ALT), aspartate aminotransferase (AST), and lactate dehydrogenase (LDH) (Figure S4A). None of these enzymes were elevated in mice with small livers (liver-to-kidney ratio < 3.08). Additionally, no cholestasis or fibrosis of the liver was observed in the affected mice, at least by macroscopic observation and H&E staining (Figure S4B,C). Regarding kidney weight, there was a sex difference in that those in males were larger than in females (Figure 1H) and a dietary difference in that those in mice fed a normal diet were larger than those in mice fed a high-fat diet (Figure 1I). However, for the incidence of hydronephrosis, defined as a liver-to-kidney ratio <3.08 and high blood ammonia levels, sex difference was consistent in that males were higher than females (Figure 3), but there was no difference by diet (Table S3). This suggests that hydronephrosis cannot necessarily be described by kidney weight alone. Therefore, we analyzed the expression levels of liver genes in *Tspan7* transgenic mice (Figure 5 and Figure 6). Because the incidence of a hydronephrosis-like pathology in *Tspan7* transgenic mice depended on the location of genomic mutations (Figure 3 and Table S2) assumed to have occurred during the mouse generation process, gene expression analysis was also performed for each of these genomic mutations. We found that urea cycle gene *Otc*, which, like *Tspan7*, is located on the X chromosome, may be involved in the onset and progression of hydronephrosis (Figure 3, Figure 4, Figure 5 and Figure 6 and Table S2) through its effect on the kidneys via the disruption of the urea cycle in the liver and subsequent ammonia accumulation. We should have conducted a similar analysis of liver gene expression in the mouse strains whose genetic predisposition would have affected the *Tspan7* transgenic mice: the background strain C57BL/6 and the *Adipoq*-Cre mouse line, a Cre driver line for the Cre/loxP system. However, mice with a liver-to-kidney weight ratio <3.08 and high blood ammonia levels were common among mice with hydronephrosis (Figure 2H,I and Figure 4B) regardless of the genomic manipulations of the *Tspan7* transgenic mice, suggesting that *Otc* may also be involved in hydronephrosis in wild-type and Cre driver mice. If so, *Otc* may be a trigger or congenital factor for hydronephrosis (Figure S5).

Notably, the impact of Cre integration on the expression of urea cycle genes, particularly *Otc*, was more pronounced in mice with mutations on the X chromosome than in those with mutations on chromosome 6 (Figure 5D), as was the incidence of hydronephrosis-like pathology (Figure 3A–D and Table S2). This indicates that the genomic mutations introduced to generate the transgenic mice promoted the development of hydronephrosis, and that mutations on the X chromosome that affected *Otc* resulted in an even higher incidence of hydronephrosis. One study indicated that *Adipoq*-Cre transgenic mice have the *Adipoq*-Cre BAC transgene inserted within the *Tbx18* gene locus on chromosome 9 [39]. Although *Tbx18* is associated with renal diseases, including congenital anomalies of the kidney and urinary tract (CAKUT) [40,41], we found no association between hydronephrosis-like pathology and *Tbx18* nor many other CAKUT-related genes when we examined renal gene expression (Figure S6). The location of the integrated foreign DNA in transgenic mice must be examined individually for each case.

The involvement of the X chromosome in hydronephrosis was further supported by the sex differences in the incidence of hydronephrosis and *Otc* gene expression. The incidence of hydronephrosis-like pathology was higher in males than in females in both *Tspan7* transgenic and Cre driver mice (Figure 3 and Figure 4, Table S2). Only the expression of *Otc* among urea cycle genes showed a significant difference between males and females (Figure 5). Furthermore, the number of X chromosome genes around *Otc* whose expression was altered by Cre integration was higher in males than in females (Figure 6). Some reports on hydronephrosis in mice have attributed the sex difference to the male hormone testosterone [42,43].

Another reason to infer X chromosome involvement in hydronephrosis is the similarity of X chromosome genomic features of patients with OTCD and the mice with hydronephrosis-like pathology examined in this study. Our data showed that in *Tspan7* transgenic mice exhibiting a hydronephrosis-like pathology, not only *Otc*, the gene most adjacent to *Tspan7*, but also other contiguous genes, *Xk*, *Dynlt3*, and *Mid1ip1*, were affected (Figure 6). These genes are also mutated in OTCD patients with clinical manifestations of nervous system disorders due to hyperammonemia. Although OTCD is considered a monogenic disease due to *OTC* mutation, patients have also been reported to have defects in multiple contiguous genes of *OTC*, including *TSPAN7*, retinitis pigmentosa GTPase regulator (*RPGR*), cytochrome b-245 beta chain (*CYBB*), *XK*, dystrophin (*DMD*), *DYNLT3*, synaptoagmin-like 5 (*SYTL5*), sushi repeat-containing protein, X-linked (*SRPX*), transmembrane protein 47 (*TMEM47*), transmembrane gamma-carboxyglutamic acid protein 1 (*PRRG1*), LanC lantibiotic synthase component C-like 3 (*LANCL3*), MID1-interating protein 1 (*MID1IP1*), and chromosome X open reading frame 27 (*CXorf27*) [35,36,37,38,44,45]. Therefore, we can speculate that in the genome of *Tspan7* transgenic mice, there may also be mutations in *Otc*, resulting in a hyperammonemic phenotype, as seen in OTCD patients. In fact, in this study, a significant number of *Tspan7* transgenic mice were observed to have high blood ammonia levels.

In certain monogenic diseases, mutations occur in *OTC* alongside the primary causative gene. Examples include granulomatous disease (OMIM 306400) due to *CYBB* mutation, Duchenne muscular dystrophy (OMIM 310200) due to *DMD* mutation [46], and McLeod syndrome (OMIM 300842) due to *XK* mutation. McLeod syndrome primarily manifests hematological symptoms such as erythrocyte abnormalities and anemia, but some patients also exhibit neurological problems such as motor disorders, cognitive decline and mental disorders, and muscular atrophy. Given that some patients with McLeod syndrome harbor mutations in *OTC* and *DMD* [47], the secondary pathology may arise from mutations in genes contiguous to the primary causative gene. Therefore, even in monogenic diseases, a single mutation may affect multiple genes in contiguous genomic regions, leading to the simultaneous occurrence of diverse clinical features, akin to a contiguous gene syndrome. The function of *Tspan7* is not well understood; however, as TSPAN7 is highly expressed in the brain, some studies using genetically engineered mice have suggested that it is involved in X-linked intellectual disabilities [48,49]. This neurological symptom may result from mutations in *Otc* that may have been introduced via *Tspan7* manipulation in *Tspan7* transgenic mice.

Chromosome X is enriched in genes associated with neurological symptoms, specifically, intellectual disability [50,51], including fragile X syndrome (OMIM 300624, mutation in *FMR1*), Lesch–Nyhan syndrome (OMIM 300322, mutation in *HPRT*), Hunter syndrome (OMIM 309900, mutation in *IDS*), lissencephaly (OMIM 300067, mutation in *DCX*), McLeod syndrome, and Norrie disease (OMIM 310600, mutation in *NDP*). Genetic disorders of chromosome X that manifest with hyperammonemia include Lowe syndrome (OMIM 309000, mutations in *OCRL*), Rett syndrome (OMIM 312750, mutations in *MECP2*), Barth syndrome (OMIM 302060, mutations in *TAFAZZIN*), ataxia (OMIM 312170, mutation in *PDHA1*), and hyperglycerolemia (OMIM 307030, mutation in *GKD*). Assuming that these monogenic diseases, including OTCD, may be categorized as contiguous gene syndromes, it might be possible that the excess ammonia and associated neurotoxicity [52] stem from mutations in *OTC*, which is contiguous to the primary causative gene of these diseases. This may explain why the X chromosome harbors numerous genes linked to intellectual disability. In light of this, the hydronephrosis-like pathology we frequently observed in *Tspan7* transgenic mice may be another example of a contiguous gene syndrome. Therefore, we suggest that *Otc* mutations that cause OTC dysfunction are a congenital factor in the development of hydronephrosis. 

One concern is that, as mutations created in transgenic mice (Figure S5) are artificial rather than spontaneous, it is questionable whether this phenomenon reflects the function of the target gene and, even more so, whether it is relevant to human physiology. Nevertheless, it may suggest the need for assessing liver function in the case of unexplained hydronephrosis-like conditions.

## 4. Materials and Methods

### 4.1. Mice and Husbandary

All experimental procedures were approved by the Institutional Animal Care and Use Committee of the RIKEN Yokohama Campus and performed in accordance with the ARRIVE guidelines.

Mice were housed in separate cages, with a maximum of five mice per cage, and maintained under an alternating 12 h light/dark cycle at 23 °C. They were provided ad libitum access to food and water. The mice were fed a standard chow diet (CLEA Rodent Diet CE-2: 12% calories from fat, 59.1% from carbohydrates, and 28.8% from protein; CLEA Japan Inc., Tokyo, Japan), which was referred to as ND, or a HF diet (CLEA High Fat Diet 32 HFD32: 56.7% calories from fat, 23.1% from carbohydrates, and 20.0% from protein; CLEA Japan Inc.). Seven-week-old male C57BL/6J mice were purchased from CLEA Japan Inc.

Transgenic mice carrying *Adipoq* promoter-driven Cre recombinase (B6; FVB-Tg(Adipoq-cre)1Evdr/J, Stock#010803, Jackson Laboratories Bar Harbor, Maine, USA) were maintained by crossing heterozygous males with C57BL/6J females. The origin and genetic background of conditional knockout mice with a loxP-flanked *Tspan7* allele and conditionally *Tspan7*-overexpressing mice harboring a CAG promoter-loxP-STOP-loxP-Tspan7 construct at the ROSA26 locus were previously described [31].

Mice were euthanized at 10–50 weeks of age; their blood was collected, and the liver and kidneys were excised, weighed, and kept submerged in RNAlater solution (Thermo Fisher Scientific, Waltham, MA, USA) at 4 °C for 20 h and stored at −20 °C for a maximum of 6 months.

### 4.2. Quantitation of Blood Ammonia

Blood ammonia levels were measured using LabAssay Ammonia (FUJIFILM Wako Pure Chemical Corporation, Osaka, Japan). Blood samples were deproteinized with a deproteinizing reagent and centrifuged at 2000× *g* for 5 min. Ammonia levels in the supernatant were determined according to the manufacturer’s instructions.

### 4.3. Quantitation of Citrulline

Dissected mouse livers (250 mg) were washed and homogenized in phosphate-buffered saline (PBS) using a TissueLyser LT instrument (Qiagen, Hilden, Germany) at 50 strokes/s for 3 min. The homogenate was centrifuged at 10,000× *g*, 4 °C for 10 min. Citrulline in the supernatant (20 mg protein) was measured using a Homocitrulline/Citrulline Assay Kit (Abcam, Cambridge, MA, USA) according to the manufacturer’s instructions.

### 4.4. Histological Analysis

Tissue samples were fixed in 10% formalin (Wako) for 20 h, dehydrated in a graded series of sucrose solutions (10% and 30%), and embedded in Neg-50 medium (Epredia, Breda, the Netherlands). Sections (7 μm thick) were cut using a cryostat microtome and subsequently subjected to H&E staining (Meyer’s hematoxylin solution and 1% eosin, Muto Pure Chemicals Co., Ltd., Tokyo, Japan). Images were captured using a BZ-X710 microscope (Keyence, Osaka, Japan) and analyzed using a BZ-X Viewer v.1.3.1.1 (Keyence) and a BZ-X Analyzer v.1.3.1.1 (Keyence).

### 4.5. Tissue Preparation and RNA Isolation

Minced tissues were homogenized in Sepasol RNAI solution (Nacalai Tesque, Kyoto, Japan) using a TissueLyser LT instrument at 50 strokes/s for 5 min. Chloroform was added to the homogenate, and the mixture was vortexed and centrifuged at 14,000× *g*, 4 °C for 10 min. The RNA phase was transferred to a fresh tube, and total RNA was purified using a QIAcube and FastGene RNA Basic Kit (Nippon Genetics, Tokyo, Japan). The RNA quality was assessed using a TapeStation (Agilent Technologies, Santa Clara, CA, USA) and RNA ScreenTape (Agilent).

### 4.6. Library Construction and Sequencing

RNA-sequencing libraries were constructed using a NEBNext Ultra II RNA Library Prep Kit for Illumina (New England Biolabs, Ipswich, MA, USA). The mRNA was enriched from total RNA (250 ng) using magnetic poly-T beads. First- and second-strand cDNAs were synthesized using random hexamer primers (included in the kit), M-MuLV reverse transcriptase, DNA polymerase I, and RNase H, and overhangs were converted to blunt ends. DNA fragments were ligated with NEBNext adaptors and size-fractionated using the AMPure XP system (Beckman Coulter, Inc., Brea, CA, USA) before treatment with USER enzyme (New England Biolabs). PCR amplification was performed using universal and dual-index primers and Phusion high-fidelity DNA polymerase. The PCR products were purified using the AMPure XP system, and library quality was assessed using a TapeStation system (Agilent). Pooled libraries were sequenced on an Illumina NovaSeq 6000 to obtain 150 bp paired-end reads.

### 4.7. Read Mapping and Analysis of Differentially Expressed Genes

Sequencing reads were aligned and mapped to the genes in the reference mouse genome (UCSC mm10) and assembled into transcripts using StrandNGS (v.4.0; Strand Life Sciences, Bangalore, India). Normalized gene expression values in transcripts per kilobase million were used to compare the pairs of sample groups. The significance levels of the differences in gene expression levels among the groups were analyzed using the unpaired Mann–Whitney *U* test (45,796 genes in total, absolute fold change >1.0, and adjusted *p*-value < 0.05).

### 4.8. Statistical Analyses

Statistical analyses were performed using GraphPad Prism 8 (GraphPad Software, San Diego, CA, USA) and SPSS 29.0 software (SPSS Inc., Armonk, NY, USA). To compare the means of the two groups, we used *t*-tests. The normality and homogeneity of variance of the data were verified using the Shapiro–Wilk test and Levene’s test, respectively. If a normal distribution could not be assumed, the non-parametric Mann–Whitney *U* test was used. Welch’s correction test was applied in the cases of unequal variance. A simple linear regression analysis was performed with age as a covariate to determine the difference in tissue weight between the two groups. Spearman correlation coefficient analysis was used to assess relationships between variables. The odds ratio was calculated using a 2 × 2 contingency table to assess the association between groups. Significance levels are represented as *, *p* < 0.05, **, *p* < 0.01, and ***, *p* < 0.001; for 0.05 < *p* < 0.1, the absolute *p*-values are provided.

## 5. Conclusions

This study suggests that dysfunction of OTC, which regulates the urea cycle in the liver, may be a congenital factor causing hydronephrosis. Our data suggest that mutations in *Otc* may arise from mutations in contiguous genes on the X chromosome or may have been introduced via genomic manipulations for generating *Tspan7* transgenic mice, including the incorporation of Cre DNA cassettes, targeting of X chromosome genes, and loxP cleavage by Cre. Thus, the hydronephrosis observed in the genetically engineered mice may not represent the primary physiological function of the target gene. This underscores the importance of considering potential artifacts resulting from genomic manipulations when interpreting phenotypes to elucidate the physiological function of a target gene.

## Figures and Tables

**Table 1:** Number and incidence of mice with dilated kidneys, a small liver, and a high blood ammonia level.

**Kidney**	**Large**				**Normal**			
BW%	≥1.5907				<1.5907			
N	59				421			
%/All	12.3				87.7			
**Liver**	**Small**		**Normal**		**Small**		**Normal**	
BW%	≤4.902		>4.902		≤4.902		>4.902	
N	31		28		89		332	
%/All	6.5%		5.8%		18.5%		69.2%	
%/Kidney	52.5%		47.5%		21.1%		78.9%	
**NH_3_**	**High**	**Normal**	**High**	**Normal**	**High**	**Normal**	**High**	**Normal**
μg/mL	≥41.7	<41.7	≥41.7	<41.7	≥41.7	<41.7	≥41.7	<41.7
N	29	2	2	25	42	47	54	275
%/All	6.0%	0.4%	0.4%	5.2%	8.8%	9.8%	11.3%	57.3%
%/Kidney	49.2%	3.4%	3.4%	42.4%	10.0%	11.2%	12.8%	65.3%
%/Liver	93.5%	6.5%	7.1%	89.3%	47.2%	52.8%	16.3%	82.8%

N, number of mice; %/All, percentage of all samples (480); %/Kidney, percentage of small or normal liver to large or normal kidney; %/Liver, percentage of high or normal ammonia (NH_3_) to small or normal liver; 1.5907, upper 95% CI for the mean kidney weight (BW%) 1.3263–1.5907; 4.902, 25th percentile of liver weight (BW%); 41.7, upper 95% CI for the mean blood NH_3_ level (μg/mL) 36.21–41.72. NH_3_ data are missing for one mouse with large kidneys and a normal liver and for three mice with normal kidneys and normal livers.

## Data Availability

The sequencing data obtained in this study were deposited in the DDBJ Sequence Read Archive (DRA) under accession number DRA017381. The data are available at https://ddbj.nig.ac.jp/ (accessed on 8 February 2024).

## Supplementary Materials

The following supporting information can be downloaded at https://www.mdpi.com/article/10.3390/ijms25137203/s1.
