# Supplementary material for: Implications of Genetic Factors Underlying Mouse Hydronephrosis: Cautionary Considerations on Phenotypic Interpretation in Genetically Engineered Mice
Source: Int J Mol Sci. 2024 Jun 29;25(13):7203. doi: 10.3390/ijms25137203 (PMC11241513; doi:10.3390/ijms25137203)
Supplement: Supplementary file 1 [file ijms-25-07203-s001.zip › ijms-3046304-supplementary.pdf]

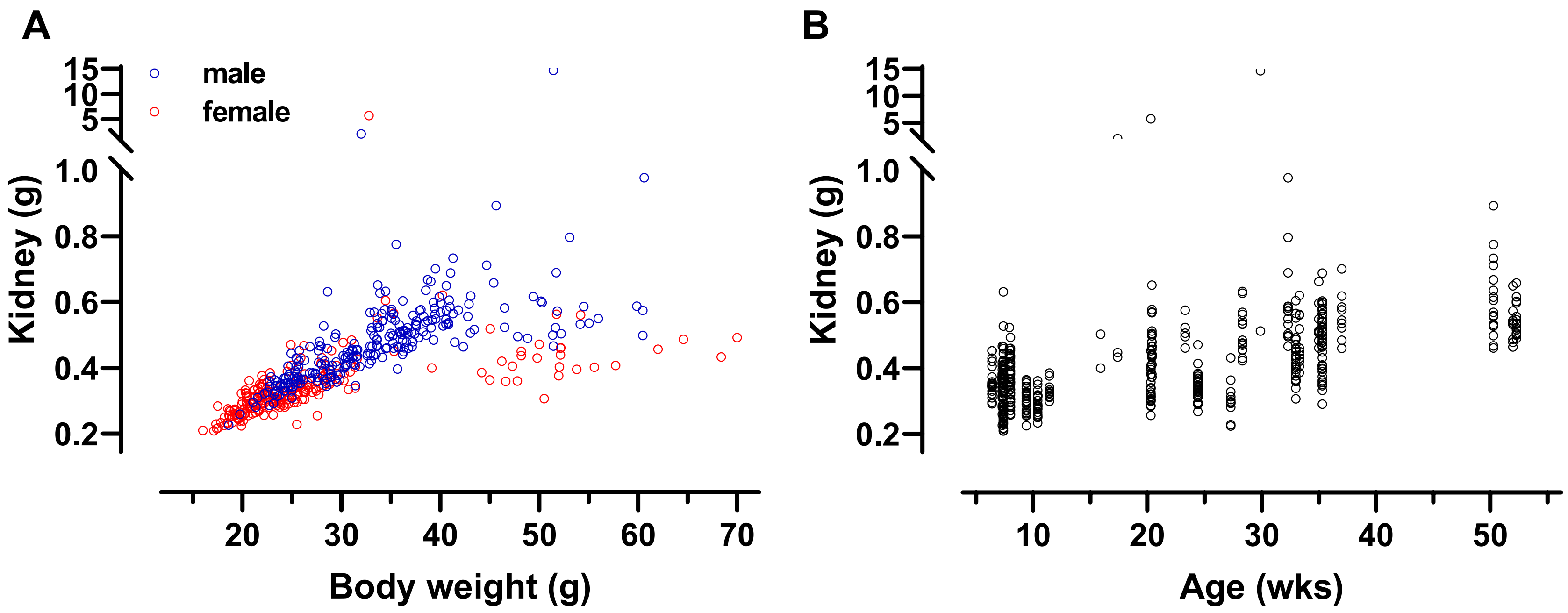

Figure S1 Kidney weight correlates with body weight. (**A**) Correlation between the absolute weight of the kidney and body weight (Spearman's test). (**B**) Distribution of the absolute weight of the kidney by age. The plot shows individual data for male and female mice from 10–52 weeks of age (n = 480).

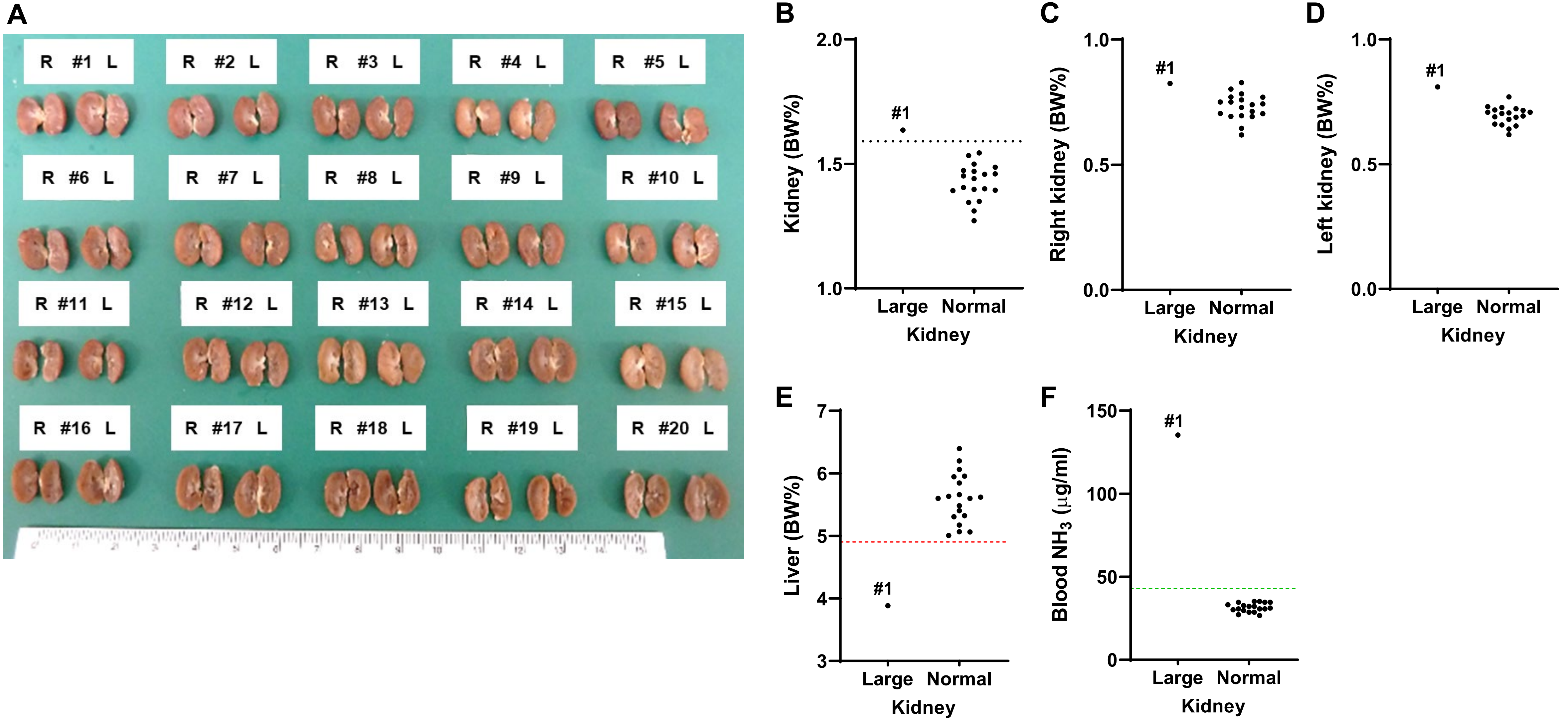

Figure S2 Characteristic phenotypes and number of mice with hydronephrosis-like pathology in intact mice. (**A**) Bisected kidneys. (**B**) Kidney weight (BW%) of 15-week-old C57BL/6J male mice (*n* = 20). The dotted line (1.5907) indicates the upper limit of the 95% CI for the mean kidney BW% in *Tspan7* transgenic mice (*n* = 480). (**C**) Right and (**D**) left kidney weights (BW%) in large and normal kidney groups of C57BL/6J male mice. (**E**) Liver weights (BW%) in large and normal kidney groups. The red line (4.902) indicates the 25th quartile of Liver weights (BW%) in *Tspan7* transgenic mice (*n* = 480). (**F**) Blood ammonia levels in large and normal kidney groups. The green dotted line (41.7) indicates the upper limit of the 95% CI for the mean ammonia concentration in *Tspan7* transgenic mice (*n* = 480). Large, mice with kidney BW% ≥ 1.5907; Normal, mice with kidney BW% < 1.5907.

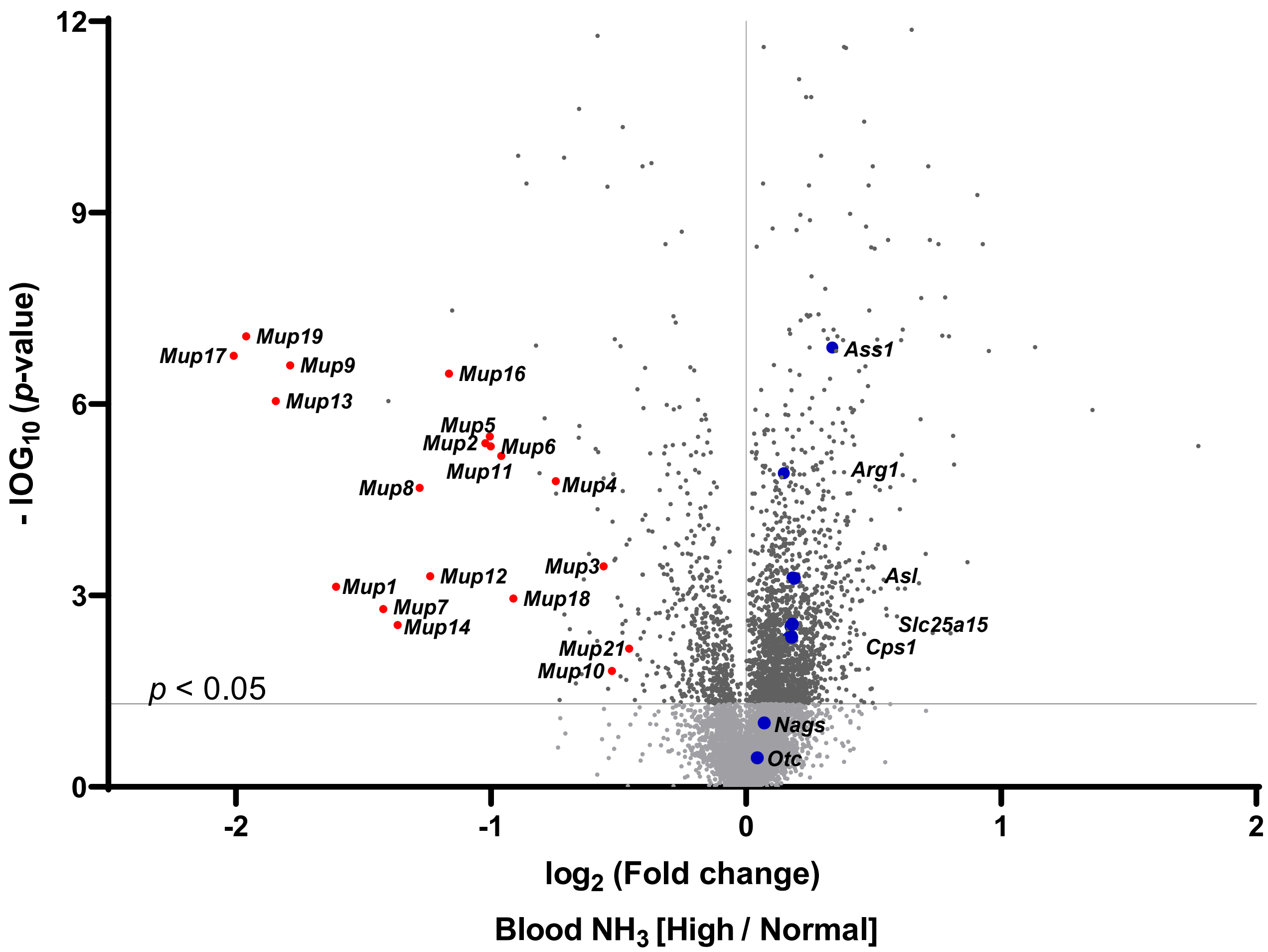

Figure S3 Volcano plot of differentially expressed genes between mice with high and normal blood ammonia levels. Each dot indicates a single gene in the liver (FC > 1, $p < 0.05$). Horizontal axis, logFC; Vertical axis, −log10 Benjamini–Hochberg-adjusted *p*-value; FC, fold change; red dots, *Mup* genes; blue dots, urea cycle genes. Blood NH_3 [High], a blood ammonia concentration ≥41.7 μg/mL.

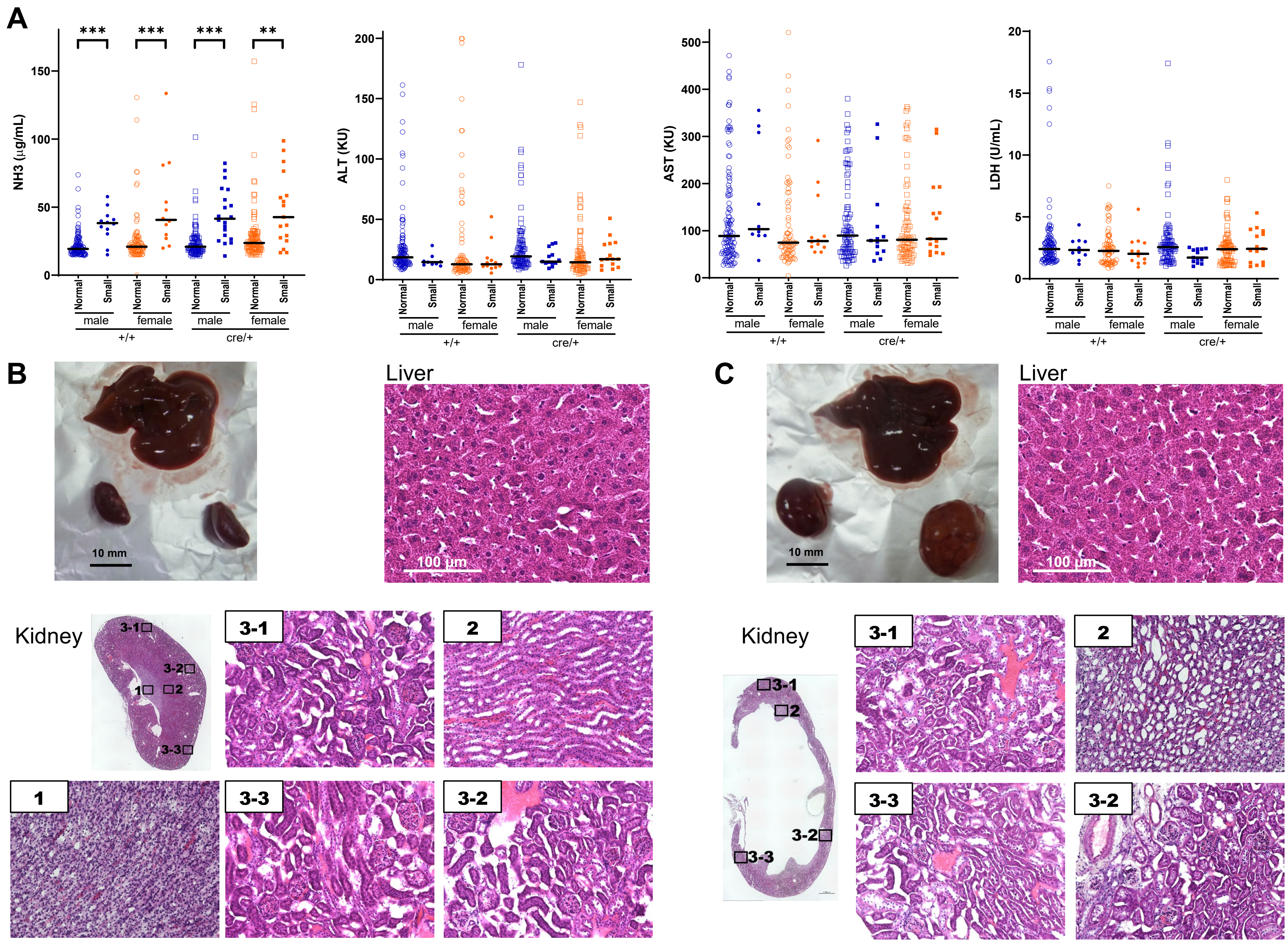

Figure S4 Liver characteristics of mice with hydronephrosis-like pathology. (**A**) Comparison of the blood levels of ammonia (NH3), alanine aminotransferase (ALT), aspartate aminotransferase (AST), and lactate dehydrogenase (LDH) between groups of mice with < 3.081 and ≥3.081 liver-to-kidney weight ratio (small and normal, respectively). The lines show medians and the plots show individual data: blue circle; male +/+ mice (*n* = 136), orange circle; female +/+ mice (*n* = 103), blue square; male cre/+ mice (*n* = 120), orange square, female cre/+ mice (*n* = 121). The statistical significance of differences among groups was assessed using the Mann–Whitney *U* test. Morphology of liver and kidney in (**B**) a control mouse and (**C**) a mouse with dilated kidneys. Top panel: liver and kidneys of a 17-week-old male mouse and hematoxylin and eosin (H&E) staining of the liver. Bottom panel: H&E staining of the right kidney. The numbers in the whole kidney images correspond to the numbers in each stained image.

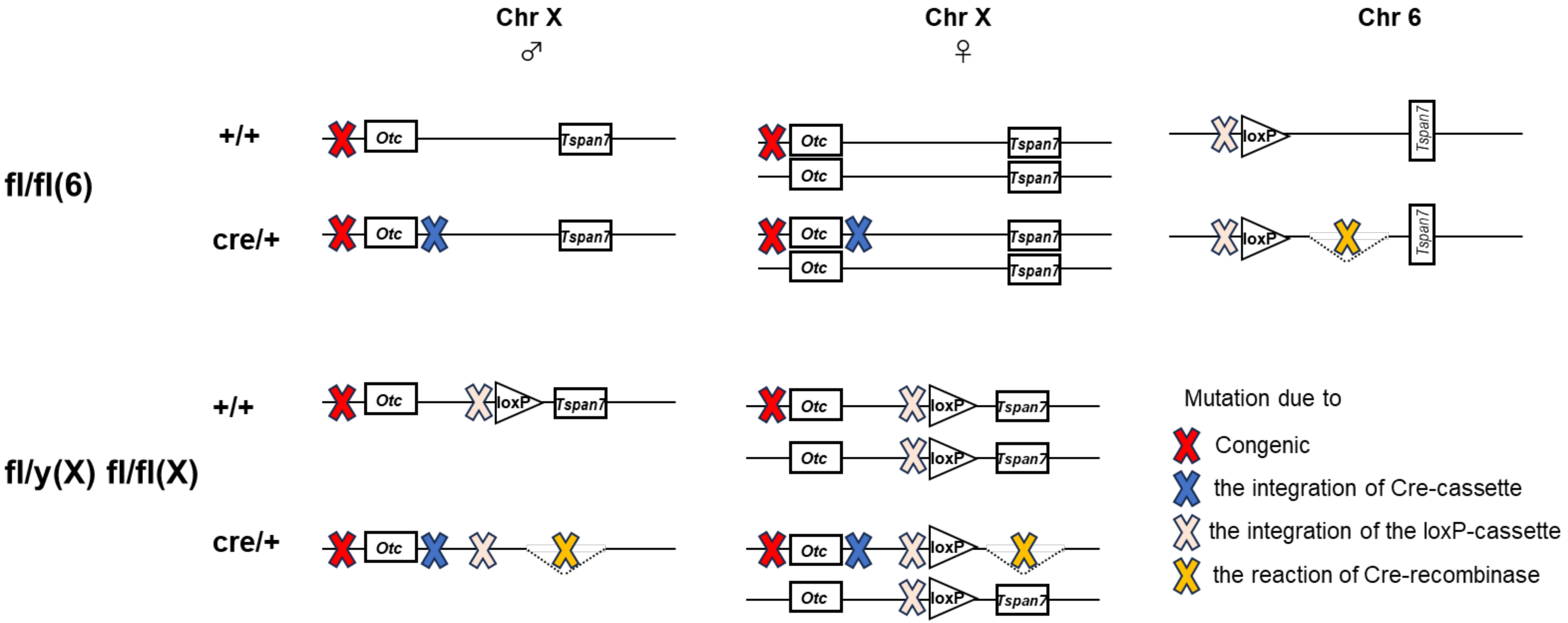

Figure S5 Hypothetical locations of mutations causing hydronephrosis. Mutations that occur during the generation of transgenic mice, including Cre recombinase DNA incorporation (blue), target gene incorporation (pink), cleavage of the loxP sequences by Cre recombinase (orange), and unknown mutation on the X chromosome (red), may trigger hydronephrosis. A mutation on the X chromosome genome around *Otc*, involved in the urea cycle, may cause the accumulation of high blood ammonia, leading to kidney dilation.

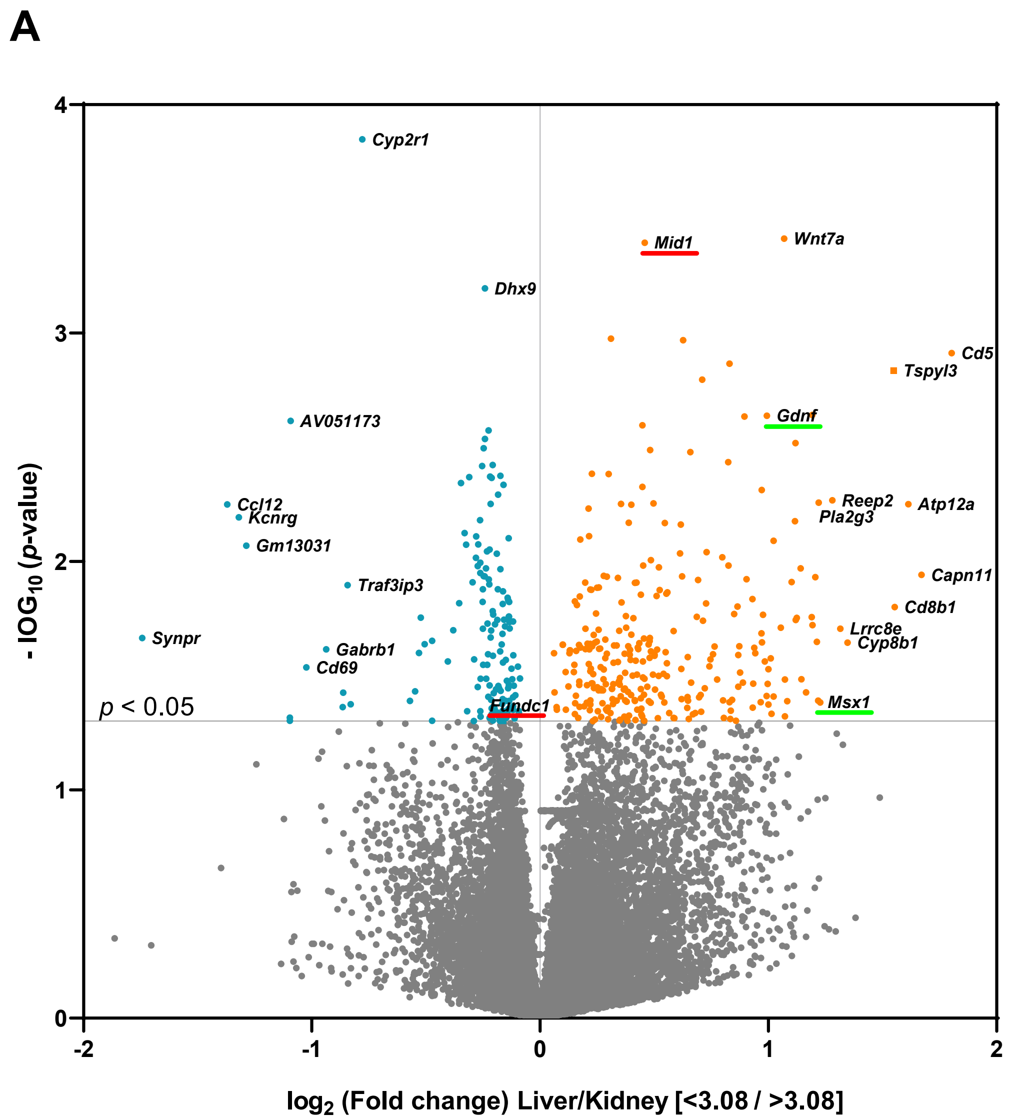

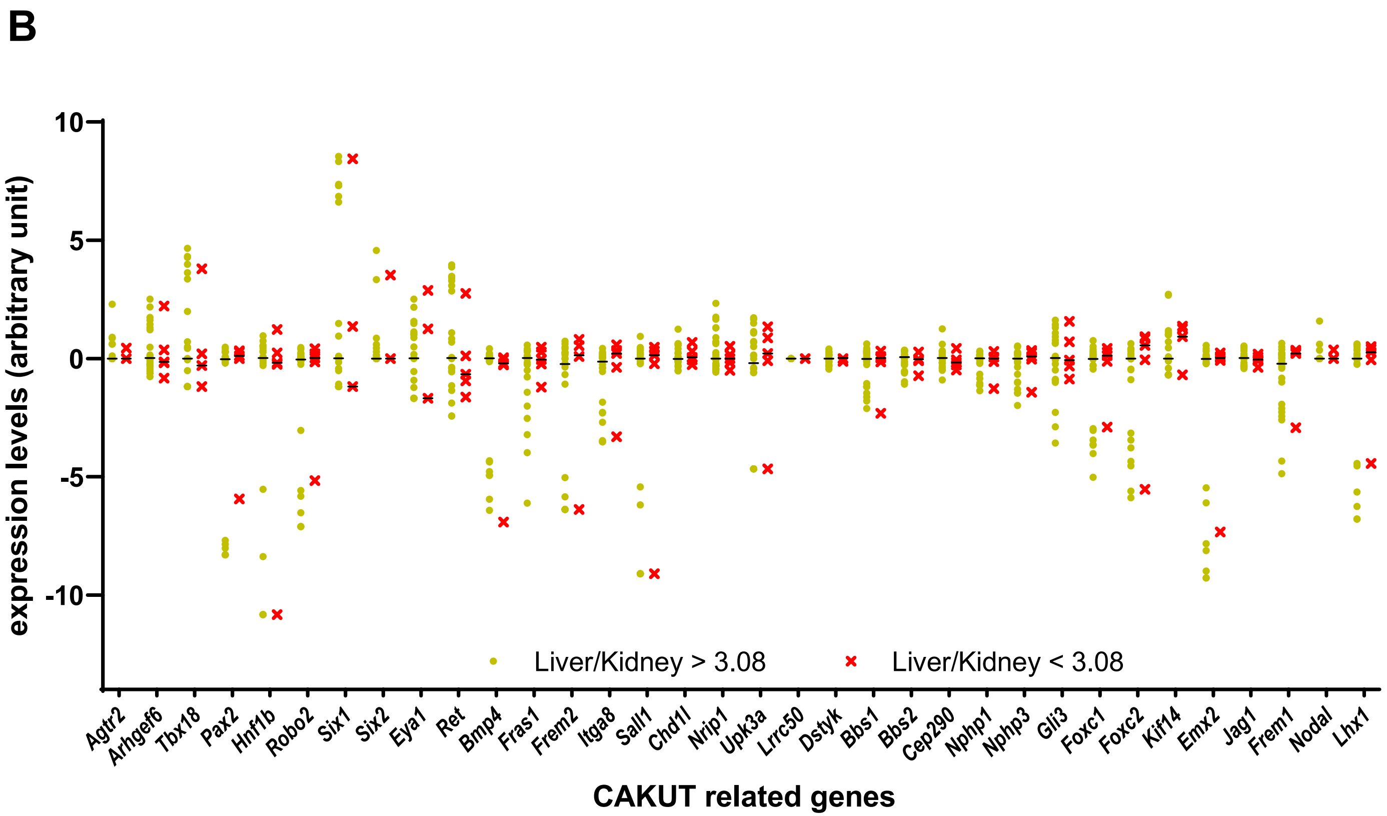

Figure S6 Kidney characteristics of mice with hydronephrosis-like pathology. (**A**) Volcano plot of differentially expressed genes between 20-week-old male mice with low (<3.081, *n* = 5) and high (>3.081, *n* = 22) liver-to-kidney weight ratio. Each dot indicates a single gene (FC > 1, $p < 0.05$). Horizontal axis, logFC; Vertical axis, −log10 Benjamini–Hochberg-adjusted *p*-value; FC, fold change; red line, X chromosome gene; green line, congenital anomalies of the kidney and urinary tract (CAKUT)-related gene. (**B**) Expression levels of CAKUT-related genes. The lines indicate medians and the plots show individual data: green, *n* = 22, liver-to-kidney weight ratio >3.081; red, *n* = 5, liver-to-kidney weight ratio <3.081. The statistical significance of differences among groups was assessed using the Mann–Whitney *U* test.

Supplementary Table S1

Odds ratio for the incidence of a small liver and large kidney in genetically modified mice.

Odds ratio	95% CI	*p*-value
7.397	1.240 - 78.69	0.0288

	Liver weight / Kidney weight		
	< 3.543	3.543 ≤	Total
Tspan7 TG	51	131	182
C57BL/6J	1	19	20
Total	51	151	202

	Liver weight / Kidney weight	
	<3.543	≥3.543
Tspan7 TG	28.0%	72.0%
C57BL/6J	5.0%	95.0%

The odds ratios for the incidence of a small liver and large kidney were calculated and compared for transgenic (*Tspan7* TG) and intact (C57BL/6J) mice. The odds ratio and *p*-value were calculated using a two-sided Fisher's exact test, and the 95% confidence interval (CI) was computed using the Baptista–Pike method. C57BL/6J, 20-week-old male mice fed a normal diet; *Tspan7* TG, 9–50-week-old male mice fed a normal diet; 3.543, the minimum value for mice belonging to the normal population in the intact mouse group (see Figure 3H).

Supplementary Table S2

Number of mice that developed hydronephrosis-like pathology and their odds ratios in mice with Cre incorporation.

			Liver/Kidney weight		NH_3 (μg/ml)		Right Kidney (BW%)	
			<3.543	Normal	>41.7	Normal	>0.745	Normal
fl/y(X)	male	cre/+	25	56	28	53	38	43
		+/+	15	73	18	67	37	51
		P	**0.046**		**0.059**		0.539	
		Odds ratio	2.173		1.966		1.218	
		95% CI	1.044–4.593		1.006–3.790		0.652–2.196	
fl/fl(X)	female	cre/+	24	60	34	50	27	57
		+/+	15	52	16	50	16	51
		P	0.456		**0.039**		0.282	
		Odds ratio	1.387		2.125		1.510	
		95% CI	0.642–3.016		1.063–4.322		0.729–3.132	

			Liver/Kidney weight		NH_3 (μg/ml)		Right Kidney (BW%)	
			<3.64	Normal	>41.7	Normal	>0.745	Normal
fl/fl(6)	male	cre/+	14	25	12	27	19	20
		+/+	8	40	8	40	13	35
		P	**0.050**		0.133		**0.046**	
		Odds ratio	2.800		2.222		2.558	
		95% CI	1.033–7.889		0.772–6.413		1.030–6.127	
	female	cre/+	8	29	8	29	6	31
		+/+	8	28	3	32	8	28
		P	> 0.999		0.345		0.564	
		Odds ratio	0.966		2.207		0.677	
		95% CI	0.333–2.808		0.649–11.20		0.194–2.007	

			Liver/Kidney weight		NH_3 (μg/ml)		Right Kidney (BW%)	
			<3.3	Normal	>41.7	Normal	>0.745	Normal
(-)	male	cre/+	10	2	5	7	11	1
		+/+	1	6	0	7	1	6
		P	**0.006**		0.106		**0.002**	
		Odds ratio	30.00		∞		66	
		95% CI	2.546–366.7		0.853–∞		3.751–789.6	
	female	cre/+	5	7	4	8	5	7
		+/+	3	4	3	4	3	4
		P	>0.999		>0.999		>0.999	
		Odds ratio	0.952		0.667		0.952	
		95% CI	0.144–5.167		0.087–3.766		0.144–5.167	

Odds ratios and *p*-values were calculated using a two-sided Fisher's exact test, and 95% CIs were computed using the Baptista–Pike method. fl/y(X) and fl/fl(X), *Tspan7*-knockout mice; fl/fl(6), *Tspan7*-overexpressing mice; cre/+, Cre-positive mice; +/+, Cre-negative mice.

Supplementary Table S3

Number of mice that developed hydronephrosis-like pathology and their odds ratios in mice fed a normal diet.

			Liver/Kidney weight		NH_3 (μg/mL)	
			<3.081	Normal	>41.7	Normal
male	+/+	ND	10	88	11	87
		HF	2	36	5	33
		P	0.509		0.771	
		Odds ratio	2.045		0.835	
		95% CI	0.512–9.673		0.290–2.299	
	cre/+	ND	20	64	19	65
		HF	1	35	5	31
		P	**0.004**		0.327	
		Odds ratio	10.94		1.812	
		95% CI	1.744–116.9		0.656–4.752	

			Liver/Kidney weight		NH_3 (μg/mL)	
			<3.081	Normal	>41.7	Normal
female	+/+	ND	10	68	13	65
		HF	2	23	5	20
		P	0.726		0.764	
		Odds ratio	1.691		0.800	
		95% CI	0.399–8.146		0.273–2.235	
	cre/+	ND	9	71	18	62
		HF	8	33	8	33
		P	0.271		0.817	
		Odds ratio	0.523		1.198	
		95% CI	0.190–1.535		0.466–3.017	

Odds ratios and *p*-values were calculated using a two-sided Fisher's exact test, and 95% CIs were computed using the Baptista–Pike method. ND, normal diet fed mice; HF, high-fat diet fed mice; cre/+, Cre-positive mice; +/+, Cre-negative mice.
